# Successful healing of aneurysmal false lumen using a second-generation drug-eluting stent in spontaneous coronary artery dissection: a case report

**DOI:** 10.1186/s43044-024-00447-9

**Published:** 2024-02-01

**Authors:** Tomiharu Niida, Kikuo Isoda, Miho Tada, Satoshi Kitahara, Yusuke Fujino

**Affiliations:** 1 Department of Cardiology, Kashiwa Kousei General Hospital, 617 Shikoda, Kashiwa, Chiba 277-8551 Japan; 2 Department of Cardiology, Juntendo University Nerima Hospital, 3-1-10 Takanodai, Nerima-ku, Tokyo, 177-8521 Japan

**Keywords:** Spontaneous coronary artery dissection, Aneurysm, Percutaneous coronary intervention, Drug-eluting stent

## Abstract

**Background:**

According to 2023 ESC Guideline, conservative medical management is generally recommended for the treatment of spontaneous coronary artery dissection (SCAD) except for patients with signs of ongoing myocardial ischemia. However, in some cases, invasive treatment (coronary artery bypass graft surgery or percutaneous coronary intervention (PCI)) is performed because of the progression of aneurysm in SCAD. Although there is no established strategy for the management of coronary aneurysm in SCAD, we report a case of successful healing of aneurysmal false lumen (AFL) using a second-generation drug-eluting stent (DES) in SCAD.

**Case presentation:**

A 44-year-old woman without any cardiovascular risk factors was transferred to our hospital due to inferior myocardial infarction. Coronary angiography (CAG) showed multiple SCADs in the coronary artery. We performed PCI to the distal right coronary artery (RCA) because the RCA showed severe stenosis (99%) with bradycardia. Six days after the first PCI, SCAD relapsed in the mid left anterior descending artery (LAD). Furthermore, AFL was observed by intravascular ultrasound imaging. To avoid enlargement of the AFL and progression of the dissection toward the proximal site of the LAD, we performed PCI to the mid LAD to seal the entry tear of the dissection using a second-generation DES. CAG revealed that the AFL in the mid LAD completely diminished at 1 year after PCI.

**Conclusions:**

The implantation of a second-generation DES might be one of therapeutic options for sealing AFL in SCAD patients.

## Background

**Fig. 1:**
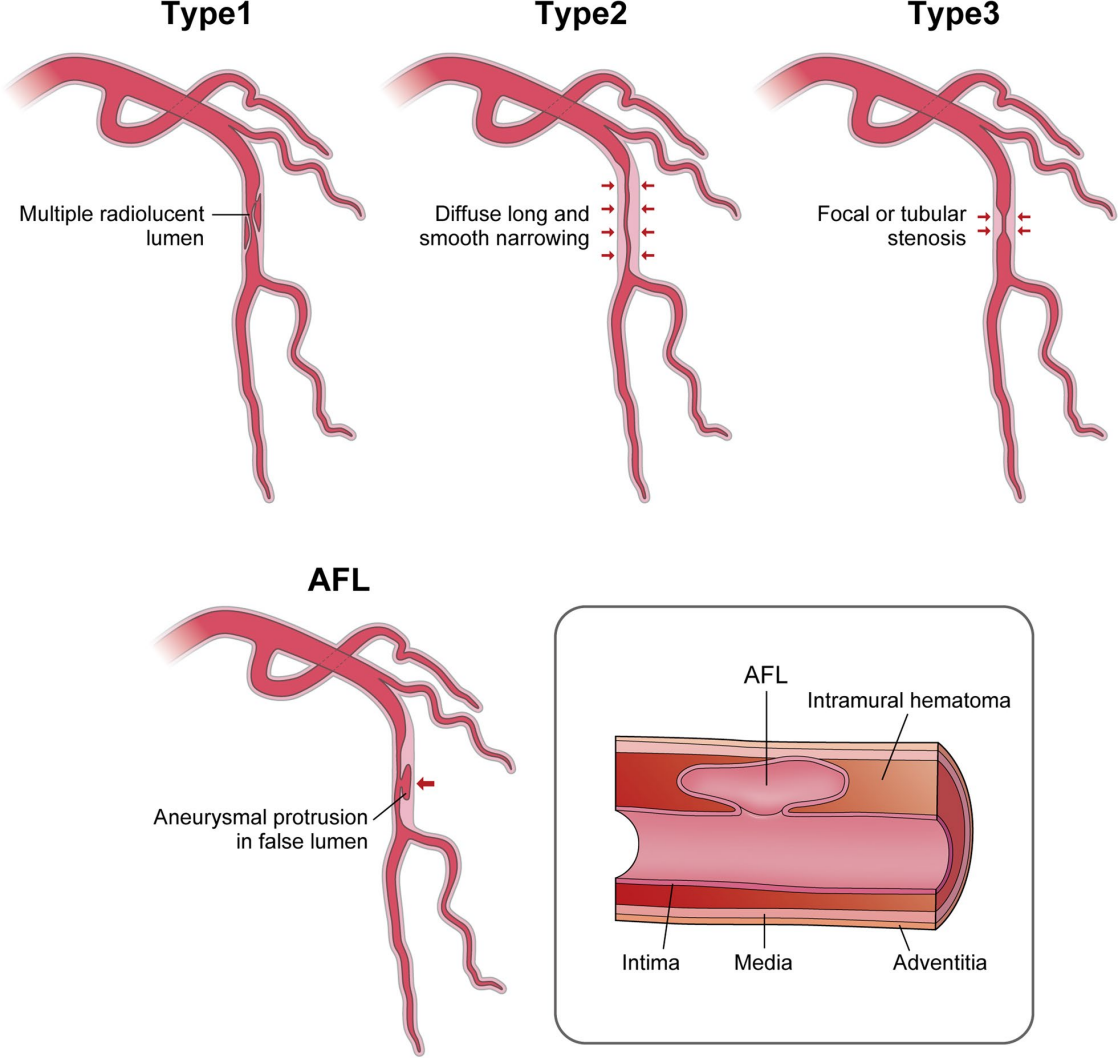
SCAD classification in angiography. Spontaneous coronary artery dissection (SCAD) is classified angiographically into 3 types. Type 1 has appearance of contrast dye staining of arterial wall with multiple radiolucent lumen. Type 2 shows diffuse long (> 20 mm) and smooth narrowing. Type 3 has focal or tubular stenosis. AFL is defined as an aneurysmal protrusion in the false lumen of SCAD. AFL: Aneurysmal false lumen, SCAD: Spontaneous coronary artery dissection

Spontaneous coronary artery dissection (SCAD) is a rare (4.0%) cause of acute coronary syndrome (ACS) in general population [1], but it accounts for 30% of ACS especially in young/middle-aged women [2]. Although SCAD is generally classified into three angiographic types [3], we rarely encounter aneurysmal false lumen (AFL) in SCAD patients as shown in Fig. 1. Coronary aneurysm in SCAD is very uncommon and there is no consensus regarding the management of SCAD aneurysm. We herein report a case of successful healing of AFL using a second-generation drug-eluting stent (DES) in SCAD.

## Case presentation

**Fig. 2:**
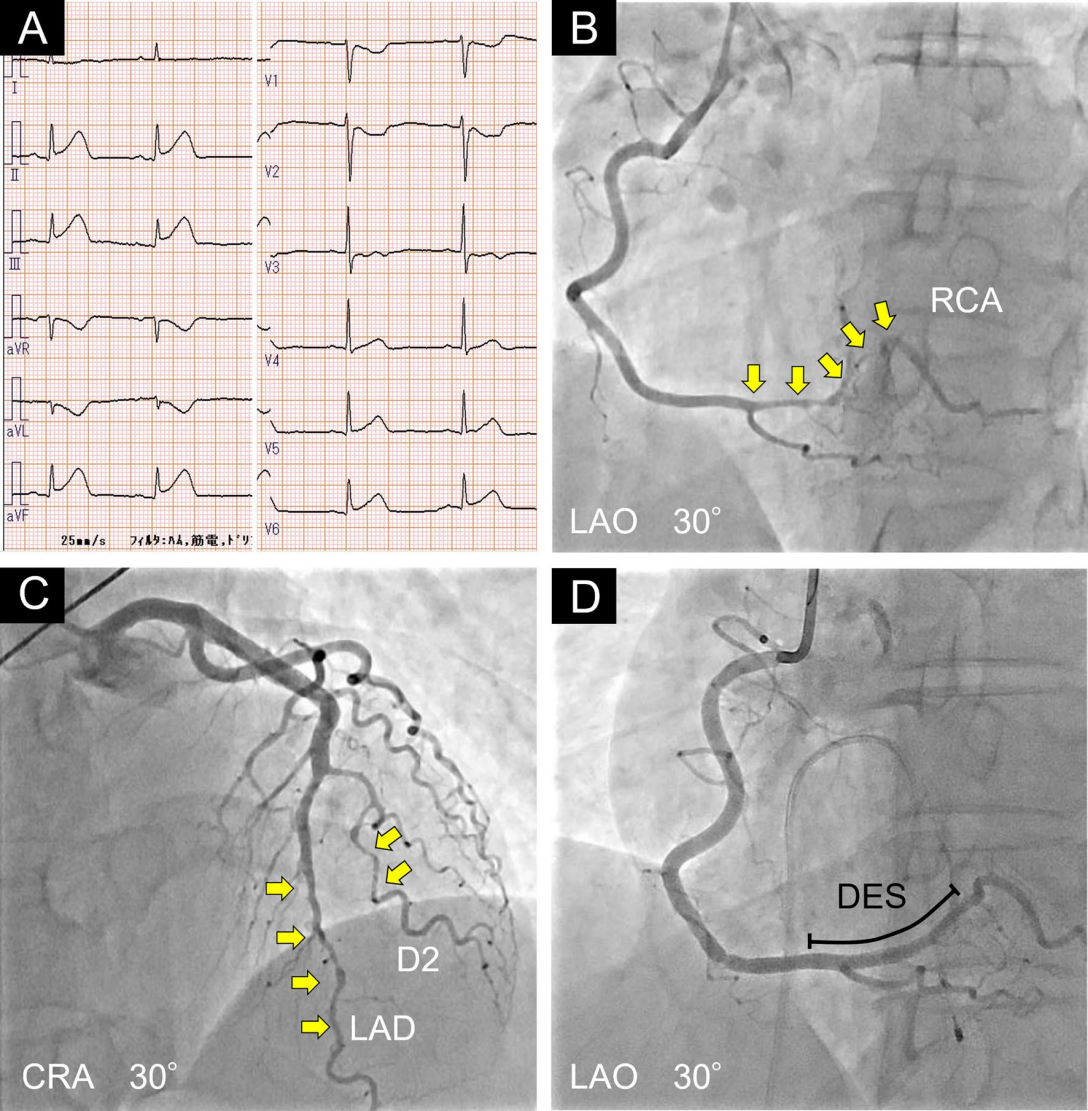
First PCI to the SCAD in the distal RCA. **A** Electrocardiogram showed ST-elevation in inferior leads (II, III and aVF). **B** The initial coronary angiography (CAG) (LAO 30°) revealed a severe diffuse stenotic lesion (Type 2) in the distal right coronary artery (RCA) (yellow arrows). **C** CAG (CRA 30°) showed moderate diffuse stenotic lesions (Type 2) in the distal left anterior descending artery (LAD) and second diagonal branch (D2) (yellow arrows). **D** Thrombolysis in Myocardial Infarction (TIMI) grade 3 flow after drug-eluting stent (DES) treatment in the distal RCA (LAO 30°). CRA: Cranial, DES: Drug-eluting stent, D2: Second diagonal branch, LAD: Left anterior descending artery, LAO: Left anterior oblique, RCA: Right coronary artery

A healthy 44-year-old premenopausal woman without any cardiovascular risk factors was transferred to our hospital due to sudden chest pain. Electrocardiogram (ECG) showed ST-elevation in inferior leads (II, III and aVF) (Fig. 2A), and non-sustained ventricular tachycardia was captured on ECG monitor. Echocardiographic examination showed severe hypokinesis in the inferior segment of the ventricle. Taken together, she was diagnosed with acute ST-elevation myocardial infarction. Emergent coronary angiography (CAG) revealed multiple SCADs in distal right coronary artery (RCA) (Fig. 2B), distal left anterior descending artery (LAD) and second diagonal branch (D2) (Fig. 2C). We decided to perform primary percutaneous coronary intervention (PCI) in the distal RCA because it showed 99% stenosis (Type 2 SCAD, Thrombolysis in Myocardial Infarction (TIMI) grade 2 flow) and the patient was hemodynamically unstable due to bradycardia. A second-generation DES (zotarolimus-eluting stent: 2.0 × 30 mm) was deployed in the distal RCA with the support of a temporary pacemaker. We added another second-generation DES (everolimus-eluting stent: 3.0 × 8 mm) in the proximal site of the first stent because persistent thrombus was observed even after repeated balloon dilatation. Finally, we achieved TIMI grade 3 flow in the RCA (Fig. 2D).

**Fig. 3:**
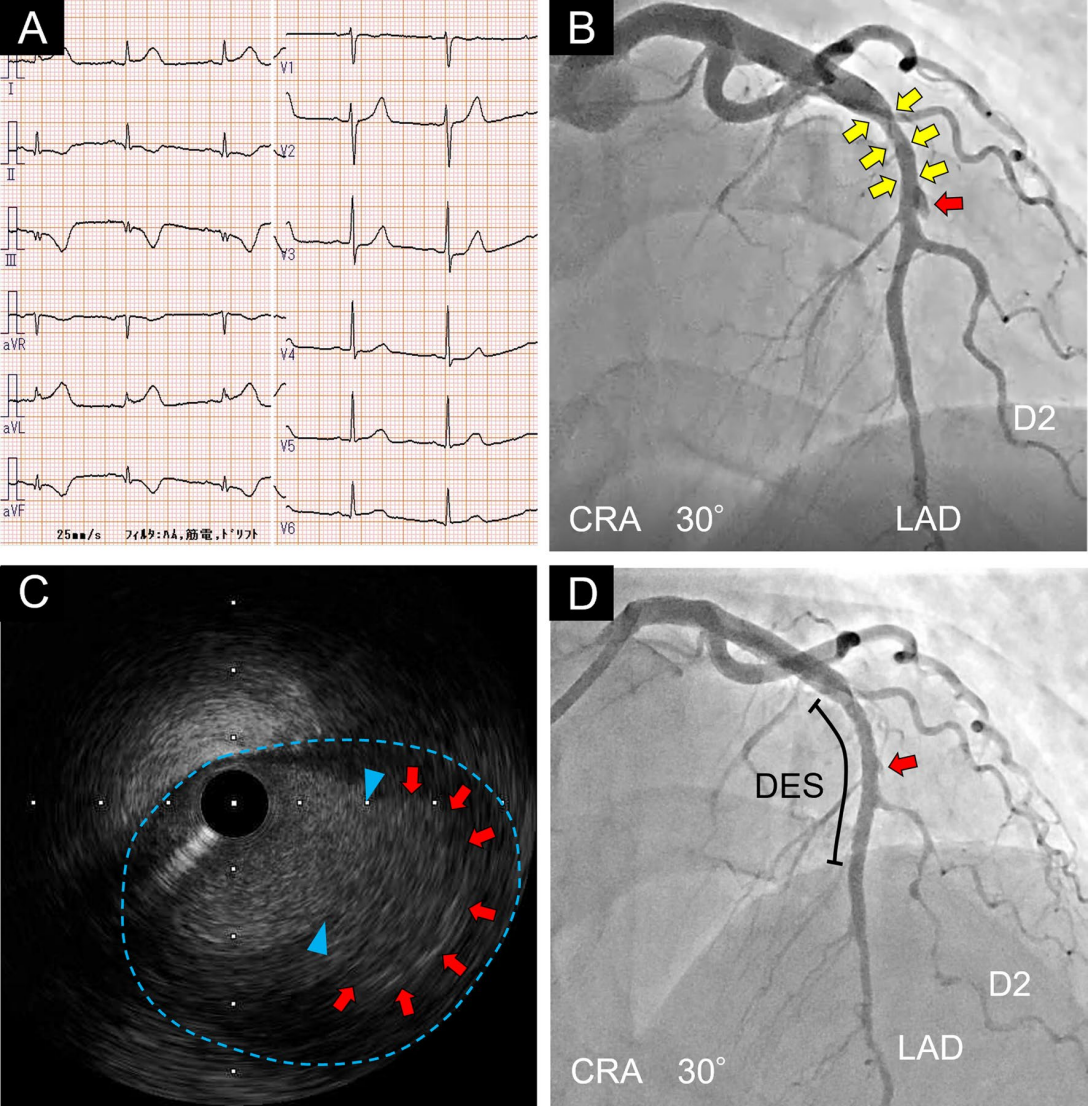
Sealing of the AFL with a second-generation DES in the mid LAD. **A**. Six days after the first percutaneous coronary intervention (PCI), electrocardiogram showed ST-elevation in lateral leads (I and aVL). **B** Coronary angiography (CRA 30°) showed a new appearance of aneurysmal protrusion in the mid left anterior descending artery (LAD) (red arrow) and elongation of the dissection to the proximal LAD (yellow arrows). **C** Intravascular ultrasound imaging of aneurysmal false lumen (AFL) in the mid LAD. Blue arrowheads indicate intimal and medial tear of the coronary artery wall. A continuous blood flow signal was observed in the AFL (red arrows). Blue dotted line indicates the adventitia of the coronary artery. **D** Blood flow in AFL decreased just after stent deployment (red arrow). CRA: Cranial, DES: Drug-eluting stent, D2: Second diagonal branch, LAD: Left anterior descending artery

**Fig. 4:**
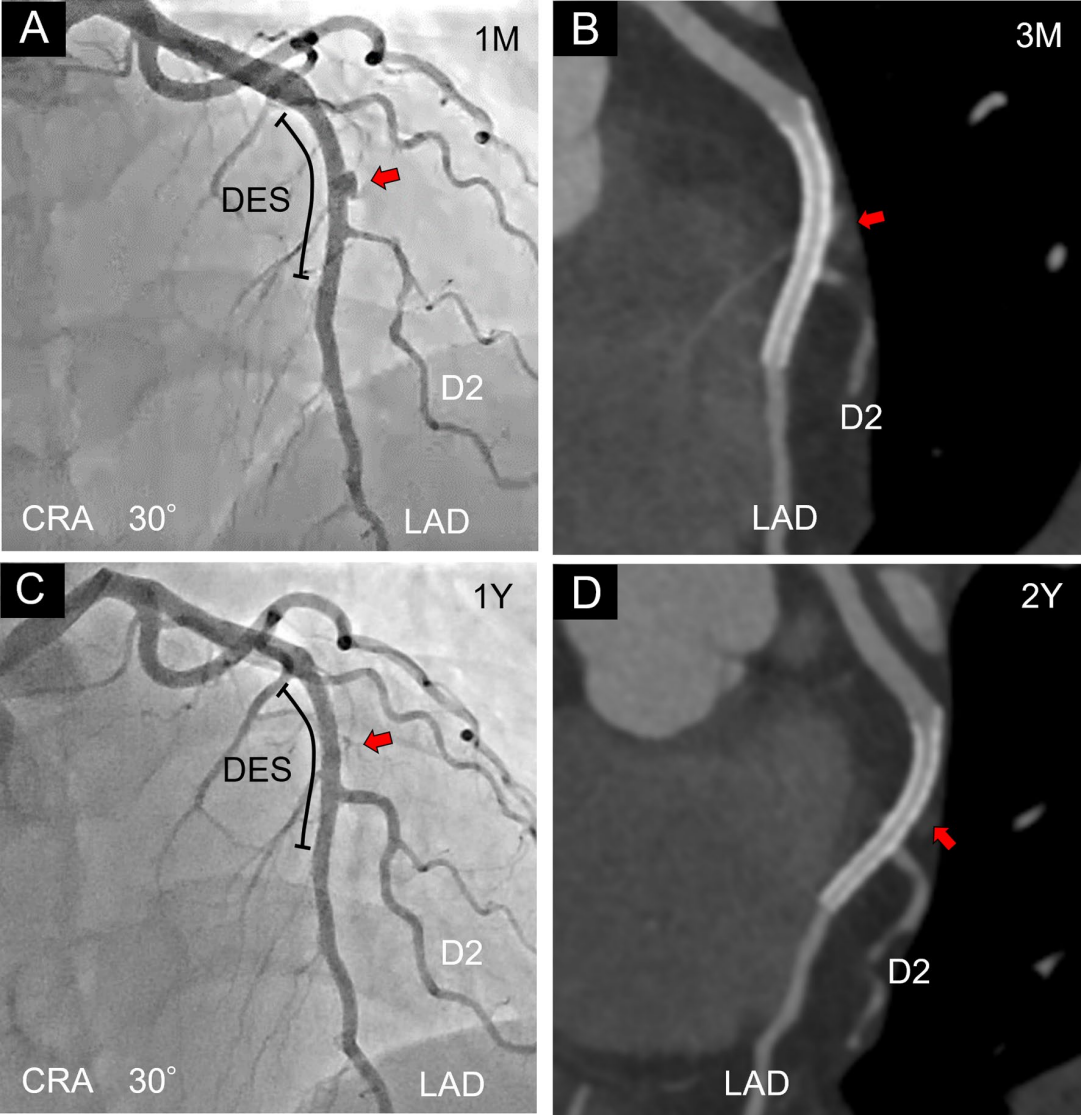
Follow-up of the AFL after PCI. **A** Coronary angiography (CAG) (CRA 30°) at 1 month after percutaneous coronary intervention (PCI) still demonstrated contrast accumulation outside the drug-eluting stent (DES) (red arrow). **B** Coronary computed tomography (CT) angiography showed decrease in size of the aneurysmal false lumen (AFL) at 3 months after PCI (red arrow). **C** One year after PCI, CAG (CRA 30°) showed the disappearance of the AFL (red arrow). **D** Coronary CT angiography revealed no contrast accumulation outside the DES (red arrow) and showed no restenosis inside it at 2 years after PCI. CRA: Cranial, DES: Drug-eluting stent, D2: Second diagonal branch, LAD: Left anterior descending artery, 1 M: 1 month, 3 M: 3 months, 1Y: 1 year, 2Y: 2 years

Six days after the first PCI, the patient complained of chest pain again and the ECG showed ST-elevation in lateral leads (I and aVL) (Fig. 3A). CAG showed a new appearance of aneurysmal protrusion in the mid LAD and a progression of the dissection to the proximal LAD (Fig. 3B). Intravascular ultrasound (IVUS) imaging revealed intimal and medial tear in the coronary artery wall. The AFL was partially occluded by thrombus; however, a continuous blood flow signal was observed in the false lumen (Fig. 3C). Considering the risk of enlargement of the AFL and distribution of the dissection toward the proximal site of the LAD, we performed PCI to the mid LAD to seal the entry tear of the dissection using a second-generation DES (everolimus-eluting stent: 3.0 × 30 mm). The blood flow in the AFL immediately decreased just after stent deployment (Fig. 3D). Although CAG at 1 month after PCI still demonstrated contrast accumulation outside the DES (Fig. 4A), coronary computed tomography (CT) angiography at 3 months after PCI showed a decrease in contrast pooling in the false lumen (Fig. 4B). We changed dual anti-platelet therapy (aspirin 100 mg/day + prasugrel 3.75 mg/day) to single anti-platelet therapy (aspirin 100 mg/day) at 6 months after PCI. CAG at 1 year demonstrated disappearance of the AFL in the mid LAD (Fig. 4C). Moreover, coronary CT angiography revealed no contrast accumulation outside the DES and showed no stent restenosis at 2 years after PCI (Fig. 4D). Our case suggests that the treatment using a second-generation DES might be one of therapeutic options for sealing AFL in SCAD patients.

## Discussion

According to 2023 ESC Guideline for the management of ACS [4], conservative medical management is generally recommended for the treatment of SCAD patients. PCI is recommended only for patients with symptoms and signs of ongoing myocardial ischemia, a large area of myocardium in jeopardy, and reduced antegrade flow. In the present case, the first PCI to RCA was necessary because of severe stenosis in RCA with bradycardia. In the second attack of SCAD, CAG showed a new appearance of an aneurysmal protrusion in the mid LAD and IVUS imaging revealed a residual false lumen with a wide fenestration. The indication for PCI must be carefully determined in SCAD patients because major adverse cardiovascular event (MACE) rates are high (approximately 60% in 3.7 years follow-up) [5]. When we perform PCI to SCAD patients, it is very important to avoid wire migration into the false lumen. Valappil et al. reported that using a floppy wire as a first wire allowed for successful PCI in 71.4% of SCAD cases [6]. Additionally, decision of stent size and length is also important. It is recommended that 5–10 mm longer stent from the margins of the dissection should be chosen to prevent extension of the hematoma [6]. The use of IVUS and optical coherence tomography (OCT) can help select the appropriate size and length of the stent. In this case, we used IVUS because OCT needs high pressure contrast injection to acquire clear images. Based on the IVUS findings, we decided to perform PCI to avoid enlargement of the AFL and progression of the dissection toward the proximal site of the LAD. Coronary aneurysm (including pseudoaneurysm) in SCAD is a very rare condition and there is no established strategy to manage it. Moghadam et al. [7] reported that coronary artery bypass graft (CABG) surgery was preferable to treat coronary aneurysm in SCAD. Nie et al. [8] described a successful case of SCAD with pseudoaneurysm treated by stent. There is also a case report that SCAD with coronary artery aneurysm was healed by conservative medical therapy [9]. However, in several cases, invasive treatment (CABG or PCI) was done because of significant progression of the pseudoaneurysm in SCAD [10, 11]. Although conservative management might be an option in the present case, we decided to use a second-generation DES to seal the entry tear of the dissection to prevent enlargement of the AFL. Therapeutic strategies for pseudoaneurysm after coronary intervention might be helpful in the management of aneurysm in SCAD. A previous report suggests that a bare metal stent (BMS) is recommended for treating coronary pseudoaneurysms, because the OCT at 9 months follow-up showed that almost all of the stent struts were covered with neointima [12]. Although BMS might promote healing of coronary artery pseudoaneurysm, in-stent restenosis due to neointimal proliferation occurs at the rate of 20–30% [13]. In contrast, first-generation DES (such as paclitaxel-eluting stent) disturbs healing of pseudoaneurysm until 2 years due to a strong inhibitory effect on neointimal formation [14]. In the present case, we used a second-generation DES (everolimus-eluting stent) because its safety (decrease in MACE) and efficacy (reduction in target vessel/lesion failure) has been proven in a randomized controlled trial compared to first-generation DES [15]. The coronary AFL in the present patient successfully healed at one year after PCI. Moreover, the stent did not show restenosis even 2 years after DES deployment. These findings suggest that the treatment using a second-generation DES might be an effective therapeutic option for sealing coronary AFL in SCAD patients.

## Conclusions

This case report highlighted the efficacy of the second-generation DES for sealing AFL in SCAD. Progression of the aneurysm has been observed in several SCAD cases. The implantation of a second-generation DES might be one of the therapeutic options for the treatment of AFL in SCAD patients.

## Data Availability

Not applicable.

## Abbreviations

ACS: Acute coronary syndrome
AFL: Aneurysmal false lumen
BMS: Bare metal stent
CABG: Coronary artery bypass graft
CAG: Coronary angiography
CRA: Cranial
CT: Computed tomography
DES: Drug-eluting stent
D2: Second diagonal branch
ECG: Electrocardiogram
IVUS: Intravascular ultrasound
LAD: Left anterior descending artery
LAO: Left anterior oblique
MACE: Major adverse cardiovascular event
OCT: Optical coherence tomography
PCI: Percutaneous coronary intervention
RCA: Right coronary artery
SCAD: Spontaneous coronary artery dissection
TIMI: Thrombolysis in myocardial infarction

## References

[1] Nishiguchi T, Tanaka A, Ozaki Y, Taruya A, Fukuda S, Taguchi H (2016). Prevalence of spontaneous coronary artery dissection in patients with acute coronary syndrome. Eur Heart J Acute Cardiovasc Care.

[2] Saw J, Aymong E, Mancini GBJ, Sedlak T, Starovoytov A, Ricci D (2014). Nonatherosclerotic coronary artery disease in young women. Can J Cardiol.

[3] Saw J, Humphries K, Aymong E, Sedlak T, Prakash R, Starovoytov A (2017). Spontaneous coronary artery dissection: clinical outcomes and risk of recurrence. J Am Coll Cardiol.

[4] Byrne RA, Rossello X, Coughlan JJ, Barbato E, Berry C, Chieffo A, et al (2023) 2023 ESC Guidelines for the management of acute coronary syndromes: developed by the task force on the management of acute coronary syndromes of the European society of cardiology (ESC). Eur Heart J, ehad191

[5] Hassan S, Samuel R, Starovoytov A, Lee C, Aymong E, Saw J (2021). Outcomes of percutaneous coronary intervention in patients with spontaneous coronary artery dissection. J Interv Cardiol.

[6] Valappil SP, Iype M, Viswanathan S, Koshy AG, Gupta PN, Velayudhan RV (2018). Coronary angioplasty in spontaneous coronary artery dissection-strategy and outcomes. Indian Heart J.

[7] Moghadam R, Rahman T, Reiss CK (2021). Complicated spontaneous coronary artery dissection (SCAD) culminating in aneurysm formation: coronary artery bypass graft surgery is preferable over percutaneous coronary intervention in peripartum SCAD. Cureus.

[8] Nie JG, Jian-Zeng Dong JZ (2014). Spontaneous coronary artery dissection and pseudoaneurysm: a case report. Am J Emerg Med.

[9] Subbramaniyam SD, Yousif N, Shivappa S, Noor HA, AbdulQader F (2022). Pregnancy-related spontaneous coronary artery pseudoaneurysm healed by medical treatment guided by optical coherence tomography. Heart Views.

[10] Rahman S, Abdul-Waheed M, Helmy T, Huffman LC, Koshal V, Guitron J (2009). Spontaneous left main coronary artery dissection complicated by pseudoaneurysm formation in pregnancy: role of CT coronary angiography. J Cardiothorac Surg.

[11] Chabrot P, Motreff P, Boyer L (2006). Postpartum spontaneous coronary artery dissection: a case of pseudoaneurysm evolution detected on MDCT. AJR Am J Roentgenol.

[12] Shintani Y, Kawasaki T (2012). The effects of a bare metal stent on the healing of a huge coronary pseudoaneurysm: a case report. Catheter Cardiovasc Interv.

[13] Hoffmann R, Mintz GS, Dussaillant GR, Popma JJ, Pichard AD, Satler LF (1996). Patterns and mechanisms of in-stent restenosis. A serial intravascular ultrasound study. Circulation.

[14] Chen D, Chang R, Ho AT, Frivold G, Foster G (2008). Spontaneous resolution of coronary artery pseudoaneurysm consequent to percutaneous intervention with paclitaxel-eluting stent. Tex Heart Inst J.

[15] Gada H, Kirtane AJ, Newman W, Sanz M, Hermiller JB, Kenneth W, Mahaffey KW (2013). 5-year results of a randomized comparison of XIENCE V everolimus-eluting and TAXUS paclitaxel-eluting stents: final results from the SPIRIT III trial (clinical evaluation of the XIENCE V everolimus eluting coronary stent system in the treatment of patients with de novo native coronary artery lesions). JACC Cardiovasc Interv.

